# Evaluation of Serological Diagnostic Test Systems Assessing the Immune Response to Japanese Encephalitis Vaccination

**DOI:** 10.1371/journal.pntd.0000883

**Published:** 2010-11-16

**Authors:** Nadine Litzba, Christoph S. Klade, Sabine Lederer, Matthias Niedrig

**Affiliations:** 1 Robert Koch-Institut, Centre for Biological Security (ZBS-1), Berlin, Germany; 2 Intercell AG, Vienna, Austria; 3 EUROIMMUN Medizinische Labordiagnostika AG, Lübeck, Germany; Centre for Cellular and Molecular Biology (CCMB), India

## Abstract

A new commercial anti-Japanese encephalitis virus IgM and IgG indirect immunofluorescence test (IIFT) was evaluated for the detection of the humoral immune response after Japanese encephalitis vaccination. The IgM IIFT was compared to two IgM capture ELISAs and the IgG IIFT was analysed in comparison to a plaque reduction neutralization test (PRNT50) and an IgG ELISA. Moreover, the course of the immune reaction after vaccination with an inactivated JEV vaccine was examined. For the present study 300 serum samples from different blood withdrawals from 100 persons vaccinated against Japanese encephalitis were used. For the IgM evaluation, altogether 78 PRNT50 positive samples taken 7 to 56 days after vaccination and 78 PRNT50 negative sera were analyzed with the Euroimmun anti-JEV IgM IIFT, the Panbio Japanese Encephalitis – Dengue IgM Combo ELISA and the InBios JE Detect IgM capture ELISA. For the IgG evaluation, 100 sera taken 56 days after vaccination and 100 corresponding sera taken before vaccination were tested in the PRNT50, the Euroimmun anti-JEV IgG IIFT, and the InBios JE Detect IgG ELISA. The Euroimmun IgM IIFT showed in comparison to the Panbio ELISA a specificity of 95% and a sensitivity of 86%. With respect to the InBios ELISA, the values were 100% and 83.9%, respectively. The analysis of the Euroimmun IgG IIFT performance and the PRNT50 results demonstrated a specificity of 100% and a sensitivity of 93.8%, whereas it was not possible to detect more than 6.6% of the PRNT50 positive sera as positive with the InBios JE Detect IgG ELISA. Thus, the IIFT is a valuable alternative to the established methods in detecting anti-JEV antibodies after vaccination in travellers and it might prove useful for the diagnosis of acutely infected persons.

## Author Summary

Japanese encephalitis, caused by the Japanese encephalitis virus, is the most prominent viral encephalitis in Asia. Three billion people live in endemic areas and at least 50,000 clinical cases occur each year, although reliable vaccines are available. Concerning the burden caused by this disease, more should be done to prevent it. Good and reliable diagnostics are one of the prerequisites for an effective fight against the virus, but it is nearly impossible to produce and evaluate an in-house assay according to high standard quality criteria as done for commercial tests. Only a few commercial assays are available and the thorough evaluation of these assays is of great importance for diagnostic laboratories. The sensitivity and specificity are statistical measures to assess the performance of a diagnostic assay. In this study the Euroimmun IgM indirect immunofluorescence test (IIFT) was compared to the Panbio and InBios IgM ELISAs. It showed a specificity of 95% and 100%, and a sensitivity of 86% and 93.8%, respectively. The specificity of the IgG IIFT in comparison to PRNT50 was 100% and the sensitivity was 93.8%. Overall, the IIFT showed a comparable performance and could be used as an alternative to the established assays.

## Introduction

Japanese encephalitis virus (JEV), a mosquito-borne pathogen of the genus *Flavivirus*, family *Flaviviridae*, is the main cause of viral encephalitis in Asia. Three billion people live in the endemic areas and at least 50,000 clinical Japanese encephalitis (JE) cases occur each year, which are a great burden to the affected populations [1], [2]. JEV is widespread throughout Asia up to the northern tip of Australia [3], [4] and is considered as an emerging or re-emerging virus [5]. It is transmitted by bloodsucking Culex mosquitoes - predominantly Culex tritaeniorhynchus. Most cases occur in rural areas, but transmission is also found in peri-urban and urban centres [6]. The disease can only be treated symptomatically but different vaccines are available for preventing it.

A mouse brain derived and formalin-inactivated JE vaccine (strains Nakayama and Beijing-1), produced by Asian companies, was the predominantly used vaccine for a long time. Only one of these vaccine products (JE-VAX®) was licensed in some non-endemic countries. In recent years Chinese manufacturers have developed a live attenuated and an inactivated vaccine, produced principally on primary hamster kidney cells, and of these the Chinese live attenuated SA14-14-2 vaccine has become widely used in endemic countries [7]. Additionally, other JE vaccines are under development or in the licensing process, e.g. a chimeric live attenuated vaccine based on the yellow fever virus 17D backbone. In this study the formalin-inactivated SA14-14-2 vaccine strain, cultivated on Vero cells, was used, which was recently licensed in the USA, Europe and Australia [2], [8], [9].

Besides the personal protection against mosquito bites, the CDC recommends administration of the JE vaccine to native and expatriate residents of endemic areas, and to travellers staying a month or longer in endemic, especially rural areas during the transmission season [10]. But the risk of JEV infection of persons from non-endemic regions travelling to endemic regions varies with duration, season, place and purpose of the stay, and should be assessed for each traveller individually even if only staying for a short time [11].

The clinical presentation of a JEV infection varies from non-specific febrile illness to severe meningoencephalitis, the main clinical manifestation. Only 1 in 250 to 500 infections is symptomatic. After the incubation period of 4 to 14 days the patients mostly show a rapid onset of fever, headache and gastrointestinal symptoms. Neurological symptoms develop subsequently, with a spectrum ranging from neck stiffness, stupor and impaired consciousness to seizures, parkinsonian movement disorders, convulsions, flaccid paralysis and coma. The mortality rate varies from 8 to 30%, and 30 to 50% of the survivors retain significant long-term neuropsychiatric sequelae [6], [12], [13], [14], [15].

The clinical picture of JE resembles the picture of acute encephalitis syndromes of other aetiologies and cannot be differentiated accurately [16]. The confirmatory diagnosis therefore requires antigen or antibody determination. Cultivation of JEV from blood and cerebrospinal fluid (CSF) is rarely positive as the viraemic period lasts only a few days [2], [12], thus diagnostics rely mostly on serological assays. The flavivirus group shows intense cross-reactivity which is highest on the IgG level. However IgM is relatively specific for the infecting virus [17], [18]. IgM is detectable in CSF and blood from almost all patients within 7 days after onset of disease [2].

For JE diagnostics “in-house” assays and commercial IgM capture ELISAs are used most commonly. Recently, a commercial indirect immunofluorescence test (IIFT) has become available. The method provides the opportunity to combine different substrates in form of an IIFT mosaic or profile in order to test for other pathogens relevant for differential diagnosis as well as to distinguish cross-reactive antibodies.

The aim of this study was to compare the performance of this new commercially available IIFT with other established commercial ELISAs and the “gold standard” in flavivirus diagnostics, the plaque reduction neutralization test (PRNT) in detecting the humoral immune response after immunisation with JE inactivated vaccine. Moreover, the course of specific antibodies after JE vaccination was determined using this new assay.

## Materials and Methods

### Sera panels

For this present retrospective study a serum collection was examined which was derived from a randomized controlled vaccination study performed by Intercell Biomedical Ltd., Livingston, UK. All details regarding the design of the vaccination study have been published previously [8], [19], [20]. Previous Japanese encephalitis or yellow fever vaccination was an exclusion criterion for the participants. The sera were obtained from 100 persons immunized with JEV vaccine IC51 (IXIARO®) on day 0 and day 28. The sera were collected at five different points in time: day 0 (V0), day 7 (V2), day 28 (V3), day 35 (V4) and day 56 (V5). They were shipped on dry ice and stored at −20°C until use. Altogether 300 different sera were chosen for the present study as described below:

The samples from V5 (n = 100) were analyzed for IgG with the Euroimmun anti-JEV IgG IIFT and with the InBios JE Detect IgG ELISA. For IgM altogether 78 samples from V2, V3, V4 and V5 were analyzed with the Euroimmun anti-JEV IgM IIFT, with the Panbio Japanese Encephalitis - Dengue IgM Combo ELISA and with the InBios JE Detect IgM capture ELISA.

As negative controls for the anti-JEV IgM and IgG IIFT the corresponding samples from V0 were incubated (IgM n = 49, IgG n = 100). For IgM an additional 29 samples that had been tested negative in PRNT50 were used as negative controls.

Blood donors from northern Germany were used to determine the JEV antibody prevalence in the population detectable with the Euroimmun anti-JEV IIFT. Two hundred IgM samples and 197 IgG samples from blood donors were assessed.

A panel of 20 JEV IgG IIFT positive sera from V5 and 10 JEV IgM IIFT positive sera from V3 and V4 was tested with the Flavivirus Profile 2, which allowed the cross-reactivity of anti-JEV antibodies with other flavivirus IIFT substrates to be assessed.

In order to determine the possibility of excluding cross-reactivity of anti-dengue positive sera in the JEV assays, a panel of 15 dengue virus (DENV) IgM positive sera was tested with the Euroimmun Flavivirus Profile 2 and a panel of ten dengue virus (DENV) IgM positive sera was tested in the Panbio and Inbios IgM ELISA. Additionally 20 DENV IgG positive sera were assessed in the Flavivirus Profile 2 and InBios JE Detect IgG ELISA.

The course of the IgM and IgG response after JE vaccination was analysed in all vaccinees, therefore all PRNT50 positive and selected PRNT50 negative samples were assessed in IgM and IgG IIFT. Six representative courses were chosen and used to present the data in this study.

### Ethics statement

This study was conducted according to the principles expressed in the Declaration of Helsinki. The study was approved by the Ethics Committee of the Medical Association of Hamburg, the Research Ethics Committee of Northern Ireland and the Ethics Committee of the Charité, Berlin. Written informed consent was obtained from all participants and all samples were analyzed anonymously.

### Serological tests

The PRNT50 was performed by the Intercell AG using a modification of the method described by Sukhavachana *et al.* The PRNT assay was carried out in a 24 well format. Briefly, serial 4-fold dilutions of heat-inactivated serum were incubated in a reaction volume of 1.2 mL for 1 h at 35°C with the JEV strain SA14-14-2. 250 µl of these virus dilutions were plated in triplicate onto monolayers of Vero cells, leading to 40–80 plaque forming units/well. A methylcellulose overlay was used to restrict virus spread. After 5 days of incubation at 35°C, the viral plaques were fixed, stained with crystal violet, and automatically counted (ProtoCOL HR Colony Counter, Synbiosis, Cambridge, UK). PRNT50 titres (the serum dilution giving a 50% plaque reduction compared to plaque formation in virus-only controls) were calculated using a linear regression (probit) analysis program. PRNT50 titres of≥1∶10 were rated as positive.

The commercial IIFT was produced at EUROIMMUN Medizinische Labordiagnostika AG, Lübeck, Germany, using Vero E6 cells infected with a strain originating form the JEV Nakayama strain (Genebank accession no. EF571853). The infected cells were fixed with acetone before inactivation of the virus by gamma irradiation. In contrast to conventional production methods the cells were grown initially on thin glass slides, which were cut into millimetre-sized fragments (biochips). The biochips were glued onto the reaction fields of microscope slides, offering the possibility to supplement the reaction fields with further biochip substrates. The JEV IIFT assembles one biochip of infected and one of non-infected Vero E6 cells in each reaction field.

For evaluation of the anti-JEV IIFT we examined IgM and IgG antibodies in the serum panels using the Titerplane technique as described in the instruction manual. For IgM detection, IgG and rheumatic factors were pre-absorbed with the Eurosorb reagent (EUROIMMUN Medizinische Labordiagnostika AG, Lübeck, Germany). Samples were diluted 1∶10, 1∶100 and 1∶1000. 25 µL of the dilutions were applied to the reaction fields of a reagent tray. The slides were then placed upside down into the recesses of the reagent tray, allowing all biochips to come into contact with the drops and the reactions to commence simultaneously under identical conditions without the need of a humid chamber.

The cells were incubated for 30 min at room temperature with the serum dilutions. After a washing step with PBS/Tween buffer, either fluorescein isothiocyanat-labeled anti-human-IgG or -IgM was added. Finally, the results were evaluated by fluorescence microscopy with a 200-fold magnification using a 450–490 nm excitation filter and a 515 nm blocking filter, without prior knowledge of the precharacterization of the sera in PRNT50.

According to the Euroimmun standard procedure, the fluorescence intensity was rated in levels from 0 (no fluorescence) to 5 (very strong fluorescence) and titres were determined as follows: In the 1∶10 dilution, for example, level 1 was rated as 1∶10, level 2 as 1∶32, level 3 as 1∶100 and level 4 and 5 were rated as more than 1∶100. Titres of ≥1∶10 were considered positive.

The Flavivirus Profile 2 (EUROIMMUN Medizinische Labordiagnostika AG, Lübeck, Germany) was produced using the same method as described for the anti-JEV IIFT. The test combines eight different flavivirus substrates. Five reaction fields in the upper row of a 10-field slide were equipped with four different biochips each, namely with tick-borne encephalitis, West Nile, Japanese encephalitis and yellow fever virus infected cells. Each of the five reaction fields in the bottom row was supplemented with four different biochips containing cells infected with one of the four dengue virus serotypes. Each serum dilution was incubated on these two different reaction fields and evaluated as described above for the anti-JEV IIFT. The assay can be used to determine the predominant flavivirus antibody response and presumptive infection.

The Panbio Japanese Encephalitis - Dengue IgM Combo ELISA (Panbio Diagnostics, Brisbane, Australia) is based on the IgM capture format with insect cell expressed and immunopurified JEV and dengue 1–4 antigens. The assay was performed as advised by the manufacturer. 100 µL of the 1∶100 diluted samples were incubated for 1 h at 37°C and after six washing steps 100 µL horseradish peroxidase conjugated monoclonal antibody/antigen complexes were incubated for 1 h at 37°C. The reaction was detected by TMB and read at 450 nm. Panbio Units were calculated as described in the manual and a JE/dengue ratio of the Panbio Units was determined. According to the instruction Panbio Unit >11 are positive and<9 are negative. Panbio Units between 9 and 11 were rated as equivocal and were not considered in the evaluation. This ratio gives presumptive information if the patient is infected either by JEV or dengue virus. A JE/dengue ratio of ≥1 indicates a presumptive infection with JEV and a ratio of <1 indicates a presumptive infection with DENV.

The InBios JE Detect IgG ELISA and IgM capture ELISA (InBios International Inc., Seattle, Washington, USA) were also performed as described in the manual. Both assays rely on the usage of the JE recombinant antigen (JERA), a serological marker which consists of different parts of JEV antigens. All incubation steps were performed for 1 h at 37°C. For the detection step TMB substrate was used, which was measured at 450 nm. For the IgG assay the wells were either precoated with the JERA or with a normal cell antigen (NCA) as a control. 50 µL of the 1∶300 diluted sera were applied to each well.

In the IgM-capture ELISA 50 µL of the 1∶100 diluted sera were used in each well. The wells were precoated with anti IgM antibodies. In a second step either JERA or NCA were added and detected with a JERA specific horseradish peroxidase labelled antibody.

Immune status ratio (ISR) was calculated by dividing the JERA OD with the NCA OD. IgM ISR values <6 are negative and >10.0 are positive whereas ratios between 6 and 10 are equivocal and were taken out of the evaluation. For the IgG ELISA ISR results >5.0 are rated as positive, values between 2.0 and 5.0 are equivocal and values <2 are negative.

The evaluation data of the Panbio Japanese Encephalitis - Dengue IgM Combo ELISA and the InBios JE Detect IgM capture ELISA with sera from infected persons have been published previously [21], [22].

All sera were blinded before being tested in IIFT and ELISA and the testing was performed by an experienced technician using the manufacturer's manual. Calculation of 95% confidence intervals (95% CI) was done using the Wilson method [23].

## Results

### IgM assay evaluation

For the IgM IIFT evaluation 78 PRNT50 positive samples (V2, V3, V4 or V5) were tested in the IgM IIFT, the Panbio IgM ELISA and the InBios IgM ELISA (Table 1). A negative cohort of 78 PRNT50 negative samples was used, which were all negative in the IgM IIFT (Table 1).

**Table 1:** Reactivity of IgM and IgG antibodies directed against JEV in ELISA and IIFT.

	anti-JEV IgM			anti-JEV IgG	
	Sera positive by Panbio ELISA/sera tested [Panbio Units range]	Sera positive by InBios ELISA/sera tested [ISR range]	Sera positive by Euroimmun IIFT/sera tested [titre range]	Sera positive by InBios ELISA/sera tested [ISR range]	Sera positive by Euroimmun IIFT/sera tested [titre range]
PRNT50 positive vaccinees	50/78 (64%) [0.9–55]	56/78 (72%) [1.4–59.9]	51/78 (65%) [0–320]	6/91 (7%) [0.7–12.4]	91/97 (94%) [0–1000]
PRNT50 negative vaccinees	nd	nd	0/78 (0%)	nd	0/78 (0%)
anti-DENV positive patients	1/10 (10%)+ [2.0–21.3]	5/10 (50%) [3.5–17.8]	3/15 (20%)* [0–32]	17/20 (85%) [1.4–27.1]	20/20 (100%)* [10–1000]
German blood donors	nd	nd	4/200 (2%) [0–10]	nd	8/197 (4%) [0–32]

nd: not done.

ISR: InBios immune status ratio;

**+:** calculation of JE/dengue ratio can be used to determine the presumptive infection;

*incubation on Flavivirus Profile 2 can be used to determine the presumptive infection.

In the Panbio IgM ELISA five of the 78 PRNT50 positive samples (6.4%) were rated equivocal and were therefore taken out of the evaluation. The equivocal samples had a geometric mean of the PRNT50 values of 1∶455. 61 of the 73 remaining samples showed concordant results in IIFT and ELISA (43 positive, 18 negative) whereas 12 samples differed. Five of these 12 samples showed false positive results in comparison to the Panbio ELISA, with low IIFT values from 1∶10 to 1∶32, and seven samples were false negative. The Panbio Units of these seven samples ranged from 13.8 to 24.9.

The geometric mean of the PRNT50 values for the 43 positive samples was 1∶535, for the 18 negative samples 1∶109, for the five samples positive in IIFT and negative in ELISA 1∶240 and for the seven samples negative in IIFT and positive in ELISA it was 1∶457.

Compared to the Panbio IgM ELISA the IgM IIFT showed altogether a specificity of 95% (95% CI 88.9–97.9%) and a sensitivity of 86% (95% CI 73.8–93.1%) with a positive predictive value of 89.6% (95% CI 77.8–95.5%) and a negative predictive value of 93.2% (95% CI 86.6–96.7%) (Table 2).

**Table 2:** Calculated specificity, sensitivity, positive and negative predictive values for the anti-JEV IgM IIFT in comparison to the Panbio Japanese Encephalitis - Dengue IgM Combo ELISA and PRNT50.

		Pre-characterization (Panbio Japanese Encephalitis - Dengue IgM Combo ELISA and PRNT50)	
	n = 151	positive	negative
**Euroimmun anti-JEV IgM IIFT**	positive	43	5
	negative	7	96
Specificity		95% (95% CI 88.9–97.9%)	
Sensitivity		86% (95% CI 73.8–93.1%)	
Positive predictive value		89.6% (95% CI 77.8–95.5%)	
Negative predictive value		93.2% (95% CI 86.6–96.7%)	

In the InBios IgM ELISA eight of the 78 PRNT positive samples (10.3%) could not be diagnosed due to an equivocal result and were not considered in the evaluation. The equivocal samples showed a geometric mean of the PRNT values of 1∶212. Only one sample was equivocal in both IgM ELISAs. 61 samples were concordant (47 positive, 14 negative) between the ELISA and the IIFT, whereas nine ELISA positive samples were negative in the IIFT with ISR values between 10.6 and 30.1.

The geometric mean of the PRNT50 values for the 47 positive samples was 1∶503, for the 14 negative samples 1∶108 and for the nine samples negative in IIFT and positive in ELISA it was 1∶433.

Altogether, the evaluation of the IgM IIFT with the InBios IgM ELISA demonstrated a specificity of 100% (95% CI 96–100%), a sensitivity of 83.9% (95% CI 72.2–91.3%), a positive predictive value of 100% (95% CI 92.4–100%) and a negative predictive value of 91.1% (95% CI 83.9–95.2%) (Table 3).

**Table 3:** Calculated specificity, sensitivity, positive and negative predictive values for the anti-JEV IgM IIFT in comparison to the InBios JE Detect IgM capture ELISA and PRNT50.

		Pre-characterization (InBios JE Detect IgM capture ELISA and PRNT50)	
	n = 148	positive	negative
**Euroimmun anti-JEV IgM IIFT**	positive	47	0
	negative	9	92
Specificity		100% (95% CI 96–100%)	
Sensitivity		83.9% (95% CI 72.2–91.3%)	
Positive predictive value		100% (95% CI 92.4–100%)	
Negative predictive value		91.1% (95% CI 83.9–95.2%)	

Altogether six samples, positive with both ELISAs did not reveal a positive result in IIFT. Differences in the determination of the specificity were found between both IgM IIFT evaluations. Five samples were negative in the Panbio IgM ELISA, but positive in InBios IgM ELISA and IgM IIFT.

**Figure 1:**
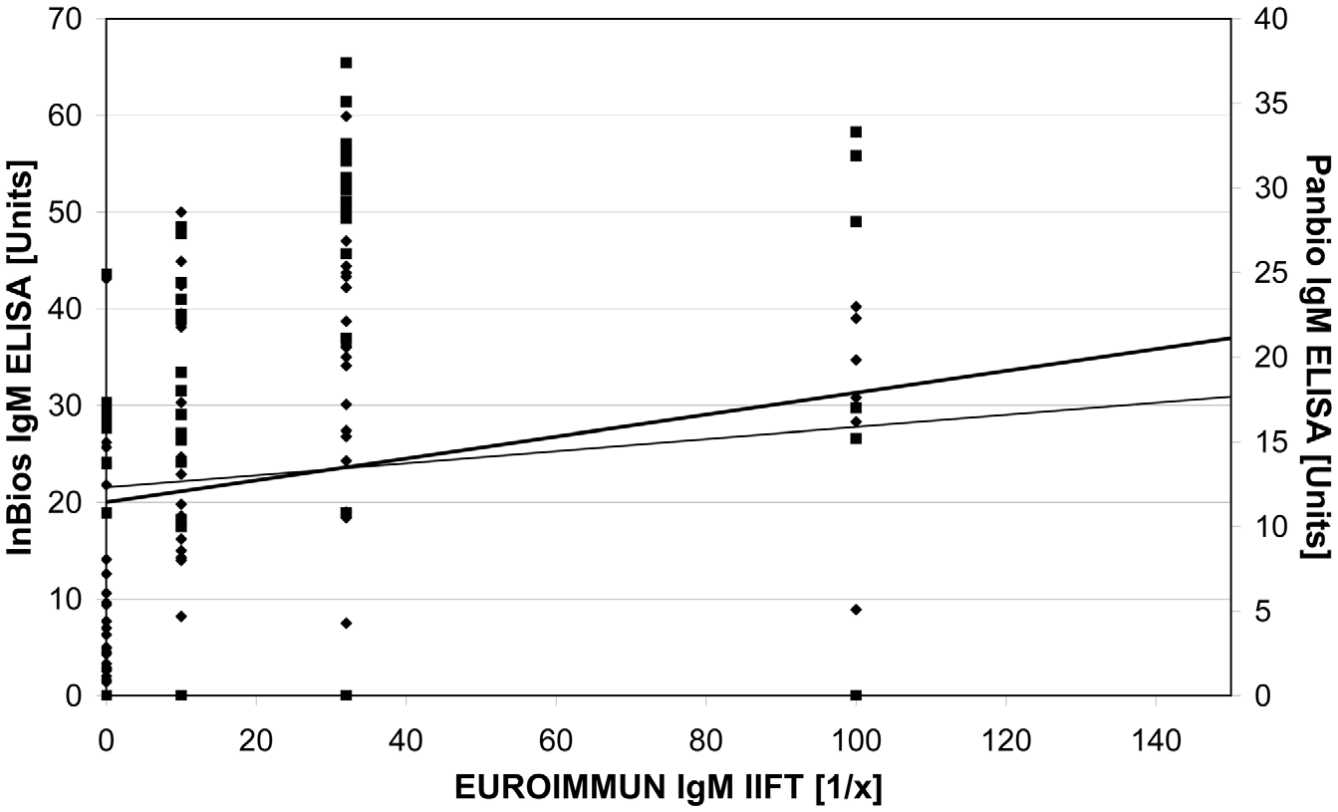
Scatter blot comparing the results of the InBios and the Panbio IgM capture ELISA with the results of the EUROIMMUN IgM IIFT. To facilitate the readability one IIFT outlier (1∶320) was taken out of the diagram. Test values: Panbio IgM capture ELISA results (▪), InBios IgM capture ELISA results (⧫). Trend line: Panbio IgM capture ELISA results (thin), InBios IgM capture ELISA results (thick).

**Figure 2:**
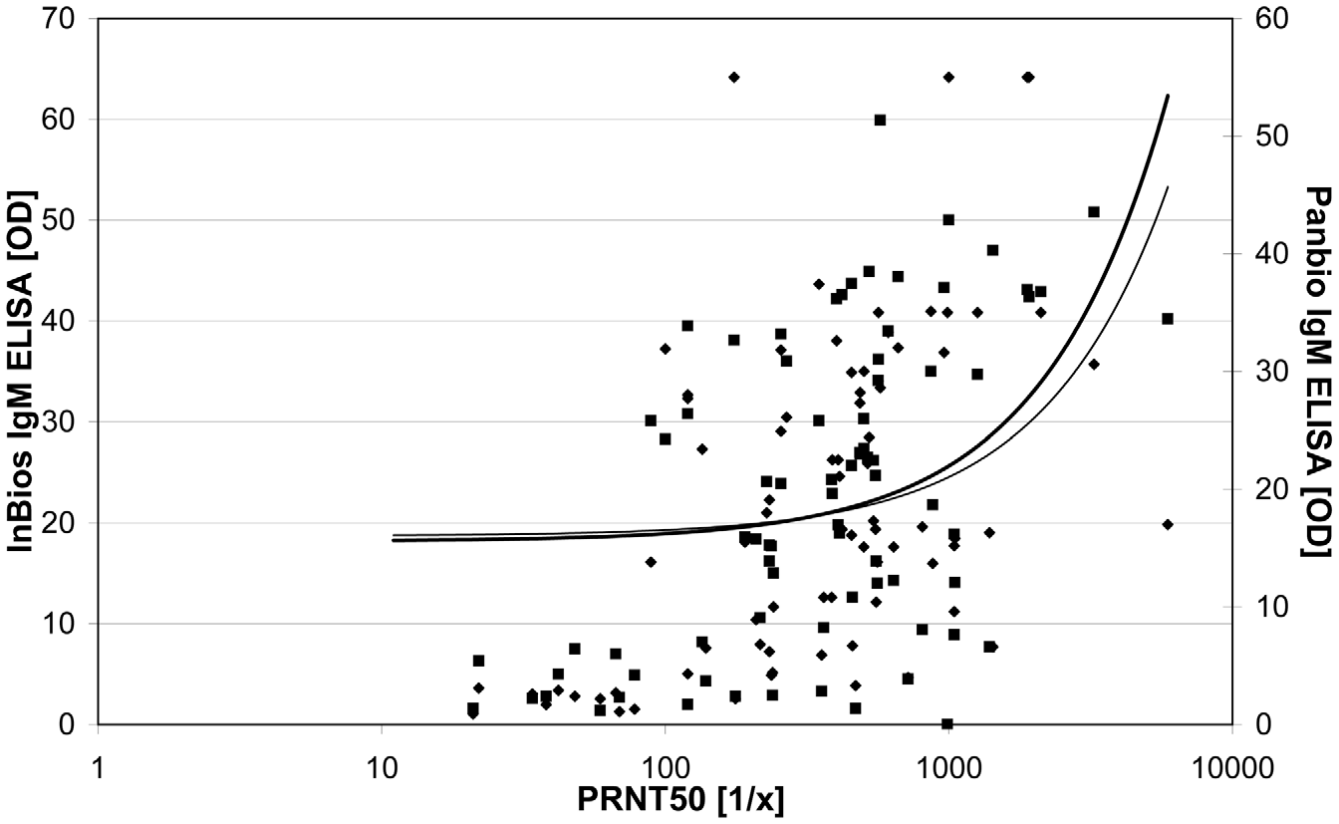
Scatter blot comparing the results of the PRNT50 with the results of both IgM ELISAs. Test values: Panbio IgM ELISA (⧫), InBios IgM ELISA (▪). Trend line: Panbio IgM ELISA (thin), InBios IgM ELISA (thick).

The comparison of the results of both ELISAs and the IIFT is demonstrated in the graphical plot (Fig. 1). The relation between the results is displayed by the increase of the trend lines. In addition, in figure 2 the OD values of both IgM ELISAs are compared to the corresponding PRNT50 results.

Additionally, the results of the PRNT50 were compared to the results of the IgM IIFT and to both IgM-capture ELISAs. In the Panbio IgM ELISA 64%, in the InBios IgM ELISA 72% and in the IgM IIFT 65% of the 78 PRNT50 positive sera were detected as positive (Table 1).

### IgG assay evaluation

**Figure 3:**
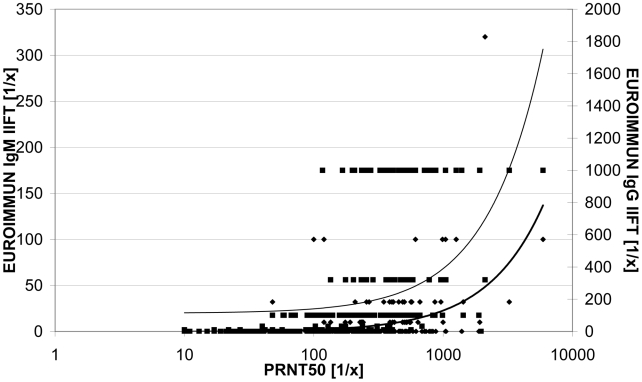
Scatter blot comparing the results of the PRNT50 with the results of the EUROIMMUN IIFT. Test values: IgM (⧫), IgG (▪). Trend line: IgM (thick), IgG (thin).

In the IgG IIFT evaluation, results of the IgG IIFT were compared to PRNT50 values of V5 (56 days post immunisation). 97 of the 100 sera showed a positive PRNT50 result. 91 of these 97 PRNT50 positive sera (93.8%) were detected as IIFT positive with titres of up to 1∶1000 whereas six PRNT50 positive sera were detected as negative by IIFT. The PRNT50 titre range of these six sera was 1∶24 to 1∶182. Two of these sera were IgG and one additionally IgM positive in the IIFT at V4, but declined to, for the IgG IIFT, undetectable levels by V5. All 100 V0 sera and the three PRNT50 negative sera of V5 were detected as negative by the IgG IIFT. In summary, the IgG IIFT showed a specificity of 100% (95% CI 96.4–100%), a sensitivity of 93.8% (95% CI 87.2–97.1%), a positive predictive value of 100% (95% CI 96–100%) and a negative predictive value of 94.5% (95% CI 88.5–97.5%) in comparison to the PRNT50 (Table 4). Figure 3 demonstrates the relation between the PRNT50 and IIFT titres. A certain distribution is detectable, but the trend line shows a clear increase.

**Table 4:** Calculated specificity, sensitivity, positive and negative predictive values for the anti-JEV IgG IIFT in comparison to the PRNT50.

		Pre-characterization (PRNT50)	
	n = 200	positive	negative
**Euroimmun anti-JEV IgG IIFT**	positive	91	0
	negative	6	103
Specificity		100% (95% CI 96.4–100%)	
Sensitivity		93.8% (95% CI 87.2–97.1%)	
Positive predictive value		100% (95% CI 96–100%)	
Negative predictive value		94.5% (95% CI 88.5–97.5%)	

Lastly, the results of the InBios IgG ELISA were compared to the results of the PRNT50 at V5. Only 6.6% (n = 6) of the InBios ELISA results were in accordance with the positive results of the PRNT50, whereas 56% (n = 51) of the positive PRNT50 results were not detected by the InBios ELISA. 37.4% (n = 34) of all 91 tested samples were equivocal and a diagnosis was not possible.

The geometric mean of the PRNT50 values for the positive samples was 1∶260, for the equivocal samples 1∶421 and for the negative samples it was 1∶198.

### Analysis of cross-reactive antibodies

The amount of cross-reactive antibodies in the anti-JEV positive samples against the different flaviviruses detectable by the Flavivirus Profile 2 IIFT was assessed. Ten JEV IgM IIFT positive sera from V3, V4 and V5 and twenty JEV IgG IIFT positive sera from V5 were tested with the Flavivirus Profile 2. In our study eight of the ten sera showed no cross-reactivity at all, two sera showed cross-reactivity against yellow fever virus, but in both cases the anti-JEV response was dominant. Cross-reactive IgG antibodies were detected in 11 of 20 anti-JEV IgG positive sera. However, again in all sera the anti-JEV response was predominant.

Moreover, a panel of 15 dengue IgM and 20 dengue IgG positive samples was tested on the Flavivirus Profile 2 to determine the possibility of differentiation between the cross-reactivity to JEV or other flaviviruses and DENV specific antibodies. On IgM level cross-reactivity against other flaviviruses was observed in four of the 15 sera. Three of these sera showed cross-reactivity against JEV. In all four samples the dengue IgM titre was higher than the titre against the other analyzed flaviviruses (three showed a difference of one and one of three fluorescence intensity stages). Ten of the DENV IgM positive sera were tested with both IgM ELISAs. All sera were diagnosed correctly with the Panbio combined JEV and DENV IgM ELISA as DENV IgM positive. In contrast the InBios IgM ELISA detected five sera as anti-JEV positive, four as equivocal and one sample as anti-JEV negative.

A cross-reactive anti-JEV IgG response was observed with the Flavivirus Profile 2 IIFT in all 20 DENV positive sera, but in 80% of the tested sera the anti-DENV IgG response was higher than the anti-JEV response (60% showed a difference of one and 20% of two or more fluorescence intensity stages). All 20 dengue IgG positive sera were tested with the InBios IgG ELISA. 85% of the sera were detected as anti-JEV IgG positive.

Furthermore, the anti-JEV antibody prevalence in the northern German population was determined by analyzing a panel of healthy German blood donors with the anti-JEV IIFT. 2% of the sera showed a positive result in the anti-JEV IgM IIFT and 4.1% in the anti-JEV IgG IIFT.

### Course of immune reaction after vaccination

The course of the IgG and IgM response after JE vaccination was analyzed additionally. All PRNT50 positive sera (n = 266) were incubated on both IIFTs. Overall 72.6% (n = 193) of these sera were detected as positive by either the IgM or IgG IIFT. IgM IIFT titres were generally lower than IgG titres. On V3 18.2% (n = 8) of the PRNT50 positive sera (n = 44) were IgG positive and 9.1% (n = 4) IgM positive, on V4 the percentage of IgG and IgM positive was 90.8% (n = 89) and 31.6% (n = 31), respectively, and on V5 it was 93.8% (n = 91) and 15.5% (n = 15).

94% (n = 94) of the vaccinated individuals (n = 100) developed within the analyzed time span a measurable IgG response, whereas only 33% (n = 33) developed an IgM response detectable with the anti-JEV IgM IIFT. Within the group of the IgM positive sera 94% (n = 31) showed detectable IgM levels at V4, whereas on V5 only 45.5% (n = 15) of the individuals were still positive.

Six vaccinees had undetectable IgG titres at all points in time. Three of these six vaccinees were PRNT50 positive for at least two points in time, two were slightly PRNT50 positive (up to 1∶54) for at least one point of time and one was PRNT50 negative. All six vaccinees showed likewise negative IgM IIFT results.

In 84.8% (n = 28) of the vaccinees with an IgM response (n = 33) IgM and IgG titres were detectable simultaneously. Only in 9.1% (n = 3) of these vaccinees the IgM reaction was detectable before the IgG response. The PRNT50 titre range of the sera undetectable in IgM IIFT was 1∶10 to 1∶1965 (n = 216, geometric mean = 133), and for the sera undetectable in IgG IIFT it was 1∶10 to 1∶317 (n = 77, geometric mean = 52).

**Figure 4:**
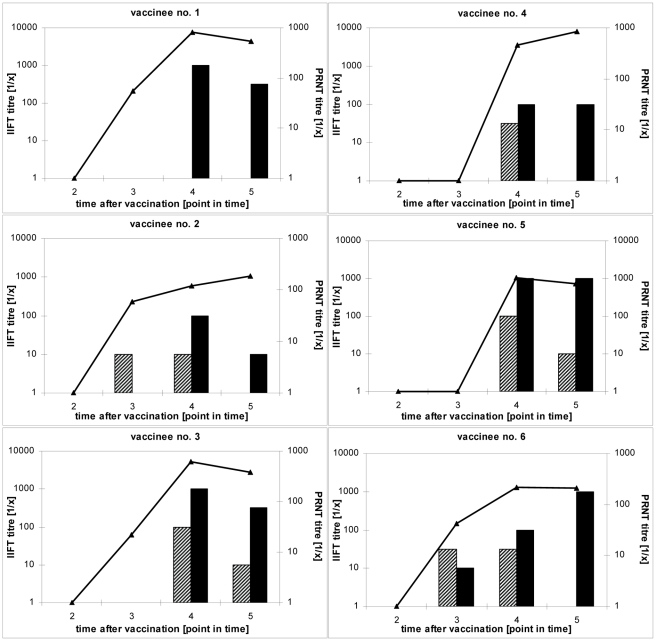
Exemplary courses of serological IgM, IgG and neutralizing antibody titres, determined by the EUROIMMUN IgM and IgG IIFT and the PRNT50. Test values: IgM IIFT titres (striped boxes), IgG IIFT titres (filled boxes), PRNT50 titres (line).

In figure 4 some typical courses of IgM and IgG titres in comparison to the PRNT50 titre are demonstrated. The course of the immune response of vaccinee no. 1 shows a clear decrease in the PRNT50 as well as in the IgG titre. As in most of the analyzed samples no IgM antibodies were detected in vaccinee no. 1. In vaccinee no. 2 both antibody classes were found. IgM is detectable before IgG as seen in 9.1% (n = 3) of the IgM positive vaccinees. Although the PRNT50 titre is increasing, the IgM and/or IgG titre measured by IIFT decreases. This effect is also seen in vaccinee no. 4 and found in 7% (n = 7) of all the vaccinees.

In 36.4% (n = 12) of all IgM positive vaccinees the IgM and IgG titres descended simultaneously, as in vaccinee no. 3. In this case the PRNT50 titre displayed the same course as the IgM and IgG titre. A simultaneous course of all measurable titres was also seen in 40% (n = 40) of the analyzed vaccinees, in 26% (n = 26) of these the PRNT50, IgM and/or IgG titres descended simultaneously and in 14% (n = 14) all measurable titres ascended simultaneously over time. Generally, in 66% (n = 66) of the vaccinees the PRNT50 titre declined towards the end of the study. In vaccinees no. 4 and 5, as in 30.3% (n = 10) of all IgM positive vaccinees, a decline of the IgM titre was observed while the IgG titre was constant until the last analysis. In 12.1% (n = 4) of all IgM positive vaccinees, as also seen in vaccinee no. 6, the IgM titre declined while the IgG titre increased over the analyzed time span.

## Discussion

The performance of the new anti-JEV IIFT was compared to two different IgM-capture ELISAs and the PRNT50.

Overall, the anti-JEV IgM IIFT showed a performance comparable to the IgM-capture ELISAs. The difference in specificity of the IIFT found in comparison to the Panbio and the concordant results compared to the InBios assay might be due to a difference in the sensitivity of the respective ELISAs. The Panbio ELISA revealed in the published evaluation study [22] with 89.3% a lower sensitivity than the InBios ELISA with 99.2%.

In direct comparison both IgM capture ELISAs were more sensitive than the IgM IIFT. This phenomenon has been detected in several IIFT evaluation studies and might be explained by different sample amounts, different antigens used for the assays and differences in the presentation of the antigen. Both ELISAs relied on recombinant antigens, whereas in the IIFT complete virus was used. However, similar numbers of PRNT50 positive samples have been detected as positive by all three methods. Thus, the IgM IIFT represents another diagnostic method which is rapid to perform and especially suitable for small serum numbers. An additional advantage of the IIFT is the possibility to identify and exclude unspecific reactive sera by recognizing the unspecific staining of the non-infected control cells.

The anti-JEV IgG IIFT revealed 56 days after vaccination a sensitivity comparable to the PRNT50, but it is much faster and easier to perform than the time-consuming PRNT50 as the performance of the IIFT takes only 1.5 hours. In contrast, the InBios IgG ELISA revealed in this study an inadequate detection of PRNT50 positive samples and is therefore not suitable to determine the antibody response after JEV vaccination.

Although the actual protective immunity can only be determined by the neutralization assay, the IgG IIFT offers a possible alternative to assess the anti-JEV IgG titre after vaccination. This might for example be a valuable tool for a rapid diagnosis of the immune status in travellers. Nevertheless, the limitation of the use of the IIFT in assessing the antibody response after JE vaccination lies in the presence of flavivirus cross-reactive antibodies from previous infections. Therefore, the intensity of the detection of cross-reactive antibodies in different serum panels was studied.

The Flavivirus Profile 2 was used to determine the level of cross-reactivity detected in IIFT in the JE-vaccinated persons. In eight of the ten sera no cross-reactivity at the IgM level was found. Similar results have been described in the literature [17]. The IgG responses however showed a high cross-reactivity, although altogether the reactivity of IgM and IgG antibodies with the other flavivirus IIFT substrates was generally lower than with the JEV substrate.

The results of the DENV positive panel tested in all three assays revealed that even the use of a recombinant antigen as found in the Panbio and InBios ELISA cannot prevent cross-reactivity. The high cross-reactivity shown by the Inbios IgM ELISA was in accordance with the results published previously [22], in which the Inbios IgM ELISA showed a specificity of 56.1%. The results of the three assays demonstrate that in areas where both viruses are endemic it is advisable to test for specific antibodies against at least JEV and DENV simultaneously [16], as performed in the Panbio assay and the Flavivirus Profile 2.

Nevertheless, to differentiate between a specific and a cross-reactive immune response in the case of an acute infection a fourfold increase in the titre of a consecutive serum should be demonstrated. However, a single IgM positive result can also be confirmed by other techniques such as PRNT. Additionally, NS1 antigen detection could be a valuable tool for the early diagnosis of Japanese encephalitis virus infections. In dengue infection e.g. NS1 is detectable very early, before IgM is produced and up to 9 days after the beginning of symptoms. The overall diagnostic sensitivity in early stages of the dengue disease was increased by the combination of NS1 and IgM detection [24].

The detection of anti-JEV IgM and IgG antibodies in the northern German population could be explained by cross-reactive antibodies against other flaviviruses, for example due to vaccination against tick-borne encephalitis or yellow fever. Therefore, also in areas without JEV and DENV occurrence it should be taken into account that cross-reactive antibodies against other flaviviruses can alter the serological results. Additionally, in patients with a relevant travel history the occurrence of dengue antibodies has to be considered.

Furthermore, the course of specific antibodies after JE vaccination detectable by IIFT was determined. Altogether only 33% of the vaccinated persons established an IgM response detectable in IIFT. Therefore, if the detection of the anti-JEV titre after vaccination is needed, it might be advisable to test for IgG rather than for IgM. Since on day 28 after vaccination the IgG response was at 18% still rather low, the best time to determine the IgG titre was from 35 days after vaccination onwards.

It is expected that in sera from acutely infected persons the fraction of sera detected as IgM and also IgG positive in the IIFT will be higher, as their serological immune response is stronger. But this has to be evaluated with a panel of sera from persons from endemic areas infected naturally with JEV.

Overall the new Euroimmun JEV IIFT showed comparable results to the commonly used commercial IgM capture ELISAs and the “gold standard” in flavivirus diagnosis, the PRNT50. It is a valuable tool for analyzing the immune response after vaccination in travellers and people resident in endemic areas and it might prove useful for the diagnosis of acutely infected persons.

## Supporting Information

Checklist S1 STARD Checklist
(0.05 MB DOC)

Flowchart S1 STARD Flowchart
(0.03 MB PDF)
