# Supplementary material for: Evaluation of Serological Diagnostic Test Systems Assessing the Immune Response to Japanese Encephalitis Vaccination
Source: PLoS Negl Trop Dis. 2010 Nov 16;4(11):e883. doi: 10.1371/journal.pntd.0000883 (PMC2982812; doi:10.1371/journal.pntd.0000883)
Supplement: Flowchart S1 — STARD Flowchart (0.03 MB PDF) [file pntd.0000883.s002.pdf]

Flowchart for the paper:

"Evaluation of serological diagnostic test systems assessing the immune response to Japanese encephalitis vaccination"

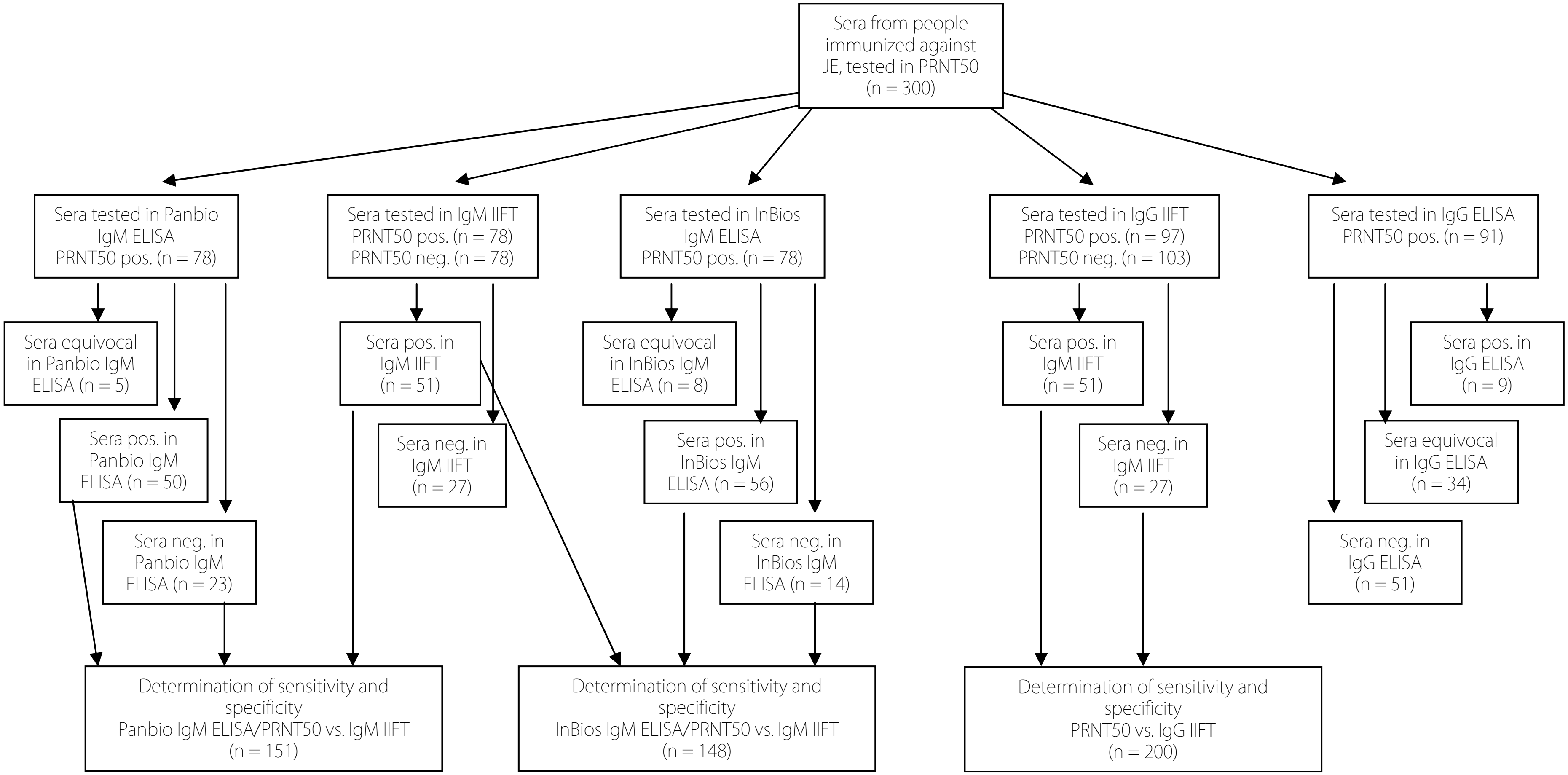

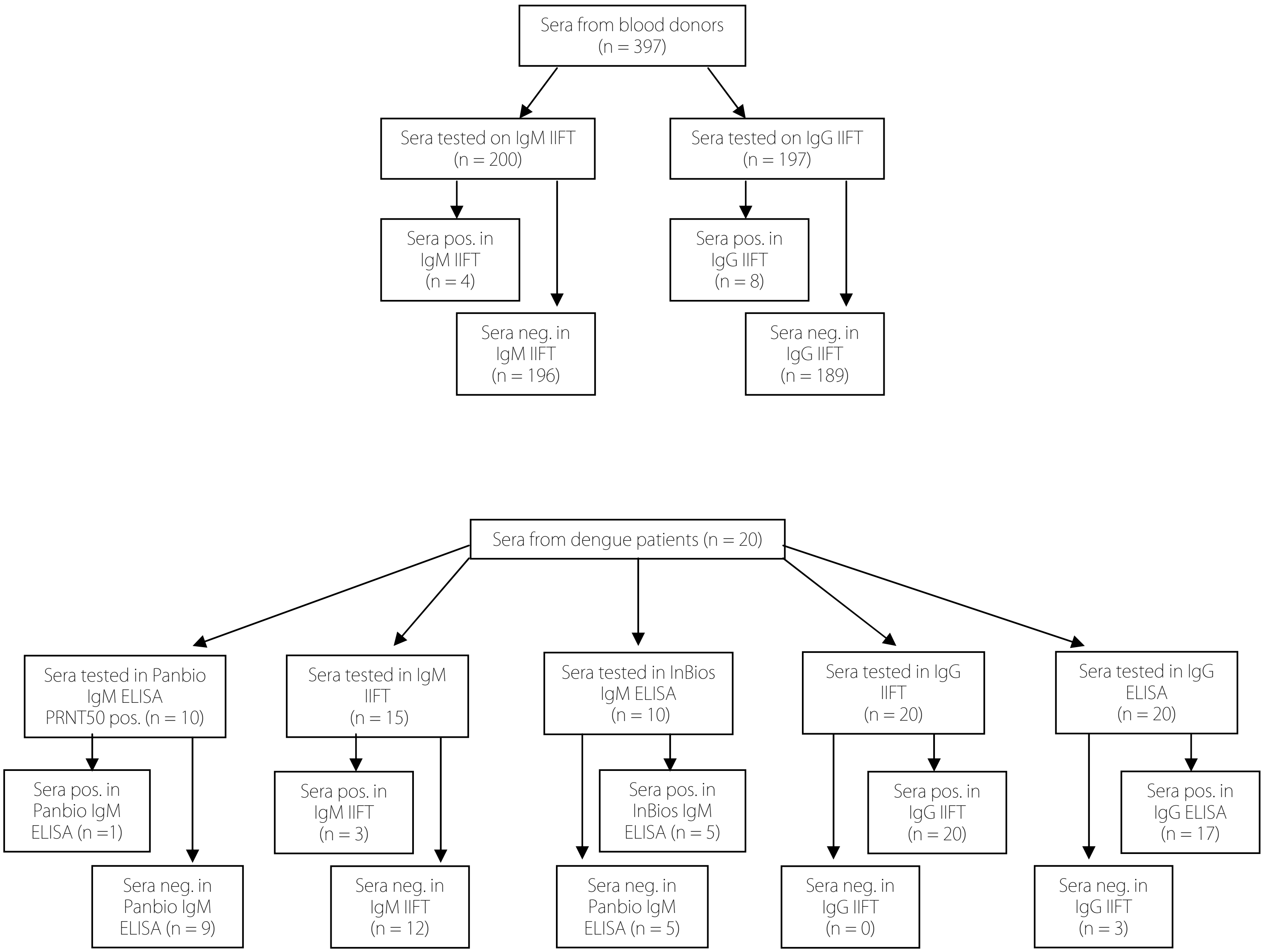
Sera from blood donors (n = 397)
Sera tested on IgM IIFT (n = 200)
Sera tested on IgG IIFT (n = 197)
Sera pos. in IgM IIFT (n = 4)
Sera neg. in IgM IIFT (n = 196)
Sera pos. in IgG IIFT (n = 8)
Sera neg. in IgG IIFT (n = 189)
Sera from dengue patients (n = 20)
Sera tested in Panbio IgM ELISA PRNT50 pos. (n = 10)
Sera tested in IgM IIFT (n = 15)
Sera tested in InBios IgM ELISA (n = 10)
Sera tested in IgG IIFT (n = 20)
Sera tested in IgG ELISA (n = 20)
Sera pos. in Panbio IgM ELISA (n =1)
Sera neg. in Panbio IgM ELISA (n = 9)
Sera pos. in IgM IIFT (n = 3)
Sera neg. in IgM IIFT (n = 12)
Sera pos. in InBios IgM ELISA (n = 5)
Sera neg. in Panbio IgM ELISA (n = 5)
Sera pos. in IgG IIFT (n = 20)
Sera neg. in IgG IIFT (n = 0)
Sera pos. in IgG ELISA (n = 17)
Sera neg. in IgG IIFT (n = 3)
